# Safety of the Oral Triphala Recipe from Acute and Chronic Toxicity Tests in Sprague-Dawley Rats

**DOI:** 10.3390/toxics10090514

**Published:** 2022-08-30

**Authors:** Warangkana Arpornchayanon, Subhawat Subhawa, Kanjana Jaijoy, Nirush Lertprasertsuk, Noppamas Soonthornchareonnon, Seewaboon Sireeratawong

**Affiliations:** 1 Department of Pharmacology, Faculty of Medicine, Chiang Mai University, Chiang Mai 50200, Thailand; 2 Clinical Research Center for Food and Herbal Product Trials and Development (CR-FAH), Faculty of Medicine, Chiang Mai University, Chiang Mai 50200, Thailand; 3 McCormick Faculty of Nursing, Payap University, Chiang Mai 50000, Thailand; 4 Department of Pathology, Faculty of Medicine, Chiang Mai University, Chiang Mai 50200, Thailand; 5 Department of Pharmacognosy, Faculty of Pharmacy, Mahidol University, Bangkok 10400, Thailand; 6 Division of Pharmacology, Department of Preclinical Science, Faculty of Medicine, Thammasat University, Pathum Thani 12120, Thailand

**Keywords:** Triphala, acute toxicity, chronic toxicity, safety, animal, regulatory toxicology

## Abstract

Background: The Indian Ayurvedic herbal formula Triphala (TPL) is known for its pharmacological properties for immunomodulation, anti-inflammation, antioxidant, and anti-cancer. This study aimed to investigate the acute and chronic toxicities of the Triphala recipe in a rat model. Methods: To assess the acute toxicities, 5000 mg/kg of TPL was orally administered to Sprague-Dawley rats. For chronic toxicities, different dose levels of TPL at 600, 1200, and 2400 mg/kg/day were given daily for 270 days. General health and behaviors and the body and organ weights of the rats were monitored. At the end of the experiment, blood samples were evaluated for hematology and biochemistry profiles. The evaluation of the internal organs’ appurtenance and necropsy was performed to confirm the tissue histopathology. Results: The results showed that there was no sign of acute toxicity in the TPL group with a decrease in sex organ weights. No significant differences in the rats’ behaviors, physical health, body, or organ weights were found between the controls and the rats receiving the 270/day of oral Triphala at 600, 1200, and 2400 mg/kg/day. However, some alterations in blood chemistries and hematology, including glucose, BUN, red blood cells, Hb, HCT, and MCV, were observed without abnormalities in histopathology. Conclusions: It has been demonstrated that the long-term use of TPL in rat models is safe. No toxic effects were found, suggesting possible safety for long-term use in humans.

## 1. Introduction

Herbal remedies have been recognized as powerful medicines and also effective methods for preserving and balancing human health since the ancient history of healthcare worldwide [1,2]. Triphala (TPL) is primarily based on the Sanskrit language, in which “Tri” means three and “Phala” means fruits. The TPL formula contains equal amounts of three dried fruits. These three different medicinal plants include *Emblica officinalis* (Family Euphorbiaceae), *Terminalia bellirica* (Family Combretaceae), and *Terminalia chebula* (Family Combretaceae) [3]. Recent reports indicate that TPL activates the immune system and enhances health and longevity [4]. It has been shown that Gallic acid and ascorbic acid are the most prevalent in TPL, suggesting an abundance of anti-oxidative effects. Accordingly, TPL can prevent radiation-induced oxidative damage [5,6,7]. Nowadays, the TPL formula is commonly used in many countries to treat various chronic illnesses such as anemia, asthma, and recurrent ulcers as a part of complementary and alternative medicines [5,6,7]. In addition to the anti-oxidant, TPL also has a protective effect against human pancreatic cancer cells in both in vitro and in vivo models [8]. 

Regardless of TPL efficiency, the safety of the continuous use of TPL is concerning. There are limited data about the long-term welfare of chronic TPL users. However, neither morbidity nor mortality is reported when a daily dose of 2,000 mg of TPL is administered for 4 weeks, in order to evaluate its effect on gut microbiota [9]. The in vitro antimutagenic activity was studied as well, and the outcome indicated that the TPL formula is safe [5,10]. The aqueous and alcoholic extracts of TPL that dose up to 1750 mg/kg reveal no signs of acute oral toxicity.

In general, toxicology tests are an important part of pharmacological research and drug development. A major purpose of testing the toxicities in animals is to establish the drug’s safety prior to clinical use in humans [11,12,13]. Usually, acute toxicity tests are conducted within the first 24 hours after a high dose of drug administration, in order to investigate any harmful effects that occur in the tested animals. Although some drugs do not exhibit major adverse effects in the course of acute toxicity testing, they may cause subacute or chronic toxicity, especially when the drugs accumulate in the internal organs at high concentrations [12,14,15,16]. Hence, chronic toxicity tests are always necessary to perform to evaluate the physiological, biochemical, hematological, and pathological safety of long-term drug use [17]. The purpose of this study was to investigate both acute and chronic toxicities of the aqueous extract of Triphala in Sprague-Dawley rats. It was the aim of this study to demonstrate the safety of TPL in animals, serving as a primary step to support the long-term clinical use of TPL in the future.

## 2. Materials and Methods

### 2.1. Plant Material and Extract

The dried fruits of *T. chebula* Retz. var. chebula. (Combretaceae) and *T. bellirica* (Gaertn.) Roxb. (Combretaceae), respectively, were obtained from Vejpong-Osot Drug Store in Bangkok, Thailand. The dried fruit of *P. emblica* L. (Euphorbiaceae) was harvested naturally in the Thai province of Nan, washed, and sun-dried for 15 days. All plants and the Triphala recipe were evaluated in accordance with Thai Herbal Pharmacopoeia methods, including organoleptic examination, % loss in drying, extractive values, total ash, and acid insoluble ash by Associate Professor Dr. Noppamas Soonthornchareonnon, Mahidol University, Bangkok, Thailand [18]. All of the dried fruits were boiled for one hour, then the process was repeated three times. After that, the residue was filtered through Whatman filter paper No.1 while the solution was spray-dried into a powder. The TPL was prepared by combining equal amounts of each powder, and it was kept in a dark bottle at 5 °C in the refrigerator until usage. Before being given to the rats, different concentrations of TPL were dissolved in distilled water.

### 2.2. Thin Layer Chromatography (TLC)

**Figure 1:**
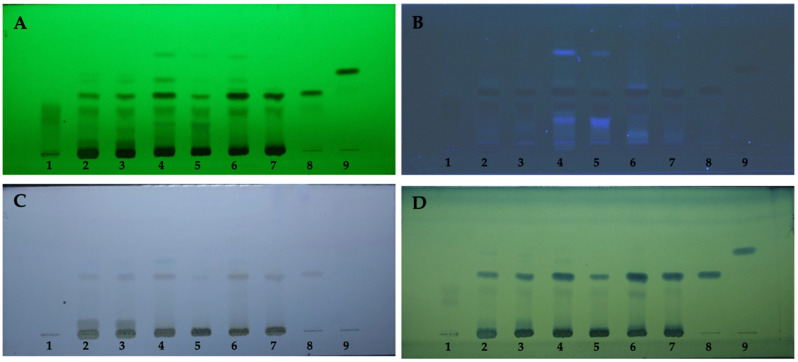
TLC chromatograms. (**A**) UV 254 nm, (**B**) UV 366 nm, (**C**) anisaldehyde/sulfuric acid, (**D**) phosphomolybdic acid; 1 = ellagic acid, 2 = *Terminalia chebula* extract, 3 = raw material of *T. chebula*, 4 = *Terminalia bellirica* extract, 5 = raw material of *T. bellirica*, 6 = *Emblica officinalis* extract, 7 = raw material of *E. officinalis*, 8 = gallic acid, 9 = protocatechuic acid.

The chemical constituents of each plant were investigated using a modified Tung et al. approach utilizing thin layer chromatography (TLC) [19]. Each extract was separated on aluminum plates precoated with silica gel 60 GF (254) (Merck, Darmstadt, Germany), and the mobile phase was toluene:ethyl acetate:formic acid (10:9:2). The reference standards were ellagic acid, gallic acid, and protocatechuic acid, which were purchased from Sigma Chemical Company (St. Louis, MO, USA). The TLC plates were dried with a hairdryer after development in the chamber, and the components were identified under 254 nm and 366 nm UV light, or the plate was sprayed with anisaldehyde-sulfuric acid or phosphomolybdic acid, as shown in Figure 1.

### 2.3. Animals

Both male and female Sprague-Dawley rats weighing between 180 and 200 g were used in this study. The room was kept at a temperature of 25 ± 1 °C with a relative humidity of 60%, and the ratio of daylight to darkness was maintained at 12-h intervals. Constant access to sufficient food and water was provided for the animals. The National Laboratory Animal Center, Mahidol University, Nakhon Pathom, Thailand, bred the rats, which were then transferred to the laboratory unit. Each rat received at least one week of care before the experiment began and they were housed at two rats per cage to avoid discomfort while still maintaining the sanctuary’s environment. The Research Ethic Committee for Animal Studies (Study code: 0004/2552), Faculty of Medicine, Thammasat University, Thailand, approved this study.

### 2.4. Acute Toxicity of Triphala 

In accordance with OECD Test Guideline 420, the initial dose of 2000 mg/kg of TPL was administered to both female and male Sprague-Dawley (SD) rats in the sighting study (Annex 2—Flow chart of the sighting research), with a report of no toxicity [20,21,22]. For the acute toxicity test, a starting dose of 5000 mg/kg was the maximum for this study [21]. Ten of each female and male rats were separated at random into control and experimental groups. All of them were fasted overnight before the experiment began. Animals in the control group received distilled water (1 mL/kg) by oral administration, while those in the test group were fed with water containing Triphala at a dose of 5000 mg/kg at a continuous volume of 1 mL/kg body weight. 

During the first 30 min and up to 24 h, any toxic indicators such as abnormal breathing, tiredness, vomiting, muscle spasticity, seizure, hematuria, and watery diarrhea would be recorded if presented [23]. The animal’s weight was assessed weekly before and after TPL administration. On the fifteenth day, the surviving animals were sacrificed with an intraperitoneal injection of pentobarbital sodium (40 mg/kg body weight). The heart, lungs, liver, kidney, spleen, adrenal glands, sex organs, and brain were removed, weighed, and grossly pathologically examined. All organs were preserved in 10% formaldehyde. 

### 2.5. Chronic Toxicity of Triphala 

The chronic toxicity test was conducted in accordance with OECD Test Guideline 452 [17]. Twenty rats, including 10 males and 10 females, were randomly and evenly distributed into 5 groups, as follows: Group 1 was administered distilled water; Group 2 was administered 600 mg/kg/day of Triphala; Group 3 was administered 1200 mg/kg/day of Triphala; Group 4 was administered 2400 mg/kg/day of Triphala; and Group 5 was administered 2400 mg/kg/day of Triphala. Only group 5, after the administration of the Triphala, was observed for 28 days before being sacrificed (Total rats = 100). 

In the test groups, each rat received the oral administration of TPL every day for 270 days. Changes in the behaviors of the animals, clinical signs, and body weights were monitored closely. Autopsies were performed on every deceased animal. On the 270th day, blood samples were taken from rats in Groups 1, 2, 3, and 4 for hematology and biochemistry evaluation. The lungs, heart, liver, kidneys, stomach, intestines, pancreas, spleen, adrenal glands, ovaries, uterus, testis, eyes, brain, spinal cords, muscles, and nerve trunks were weighted and then examined macroscopically and microscopically to identify any abnormalities. Group 5 rats were observed for an additional 28 days before blood samples and necropsies were taken and performed. 

### 2.6. Statistical Analysis

In the acute toxicity study, Student’s *t*-test was used to process the data. The chronic toxicity test was evaluated for statistical significance using one-way analysis of variance (ANOVA) and Dunnett’s test. The results were expressed as the mean ± standard error of the mean (S.E.M.). *p*-values less than 0.05 were considered significant. GraphPad Prism 8.0 software was used for all data analysis (GraphPad Software, Inc., San Diego, CA, USA). 

## 3. Results

### 3.1. Evaluation of the Acute Toxicity of Triphala 

A single oral dose of TPL at a high concentration (5000 mg/kg) did not result in mortality in both female and male SD rats. Normal patterns of behavior were observed in all groups. There were no abnormalities in animals’ eyes, skin, fur, mucous membranes, respiratory system, circulatory system, autonomic nervous system, or central nervous system. In addition, there was a non-significant difference in body and internal organ weights between the TPL-treated rats and the control group on day 14 (Table 1 and Table 2). However, a reduction in uterus and testis weights showed a significant difference from the control group. 

### 3.2. Evaluation of the Chronic Toxicity of Triphala 

There was no statistical difference in body weight when the TPL was administered at 600 mg/kg for 270 days. At 2400 mg, the body weight of female rats in the satellite group was slightly less than the controls (Table 3 and Table 4). Meanwhile, the body weight of male rats declined significantly on day 90 but increased later. The highest body weight of the male rats was seen in the TPL 1200 mg/kg group. 

Table 5 reveals that increases in weight in the heart (1200 mg/kg), liver (2400 mg/kg and satellite group), and spleen (all treatment groups) were observed, and the adrenal gland and kidney (satellite group) of female rats treated with the TPL were significantly smaller as compared to untreated rats. In male rats fed with the TPL, the liver (satellite group), pancreas (1200 mg/kg), kidneys (600, 1200 mg/kg, and satellite group), testis (1200 and 2400 mg/kg), and epididymis (2400 mg/kg) were significantly increased in weight (Table 6).

Concerning the hematological parameters in rats, the values of mean corpuscular hemoglobin (MCH) and mean corpuscular hemoglobin concentration (MCHC) were a little decreased in the females treated with 600 mg/kg of TPL, whereas the overall white blood cell count (WBC) or WBC differentiation remained unchanged (Table 7). Among the male rats, the following parameters, red blood cell count (RBC), hemoglobin (HB), hematocrit (HCT), and mean corpuscular volume (MCV), were considerably lower than those of the rats in the control group (Table 8). Noticeably, the levels of neutrophils (NEU) were significantly lowered in all of the TPL-treated groups except for the female rats.

The effects of different concentrations of TPL on organ functions are shown in Table 9. In female rats treated with Triphala, the level of blood urea nitrogen (BUN) was significantly increased compared to the control group, with the exception of the satellite group. At a dose of 2400 mg/kg, the female rats exhibited a significant increase in creatinine levels. However, all of these values were still within normal limits. In male rats, the glucose levels were higher in all treatment groups, especially at a dose of 1200 mg/kg and in the satellite group. Unlike the females, a rise in BUN level was seen only in the satellite group of males. Compared with the controls, the total protein levels were significantly lowered in the animals receiving 1200 mg/kg, 2400 mg/kg, and in the satellite group. The total bilirubin level decreased significantly in the 2400 mg/kg TPL-treated group. The aspartate transaminase (AST), alanine aminotransferase (ALT), alkaline phosphatase (ALP), and direct bilirubin levels showed no differences from the control group. 

**Figure 2:**
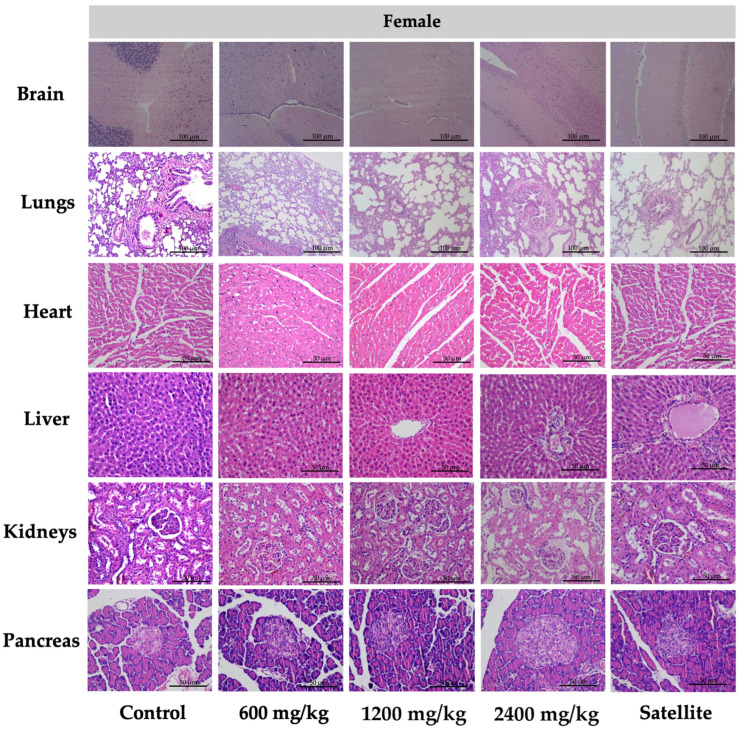
Histology of tissue from the chronic toxicity testing of Triphala in female rats. All histological sections of the brain, lungs (hematoxylin and eosin staining, 10×), heart, liver, kidneys, and pancreas (hematoxylin and eosin staining, 40×) of rats.

**Figure 3:**
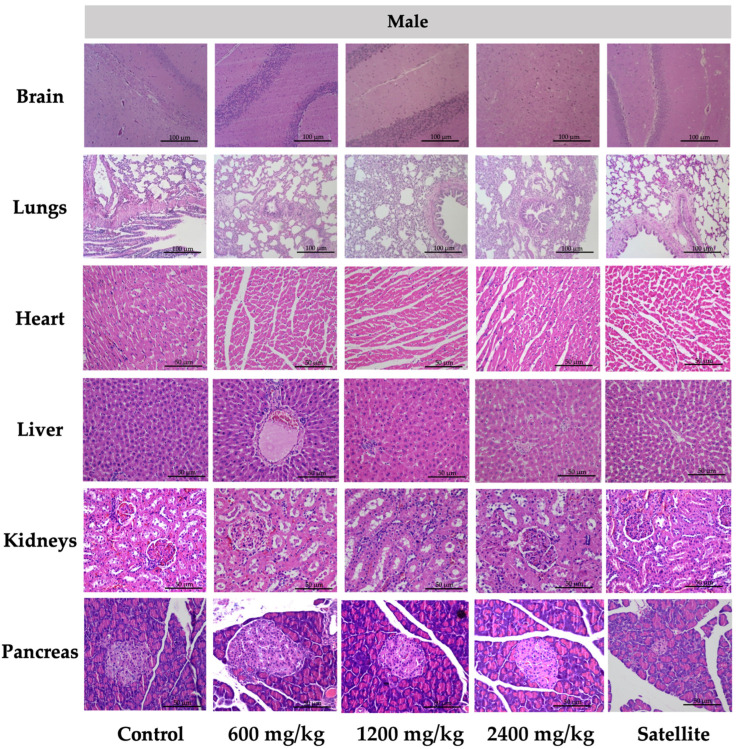
Histology of the tissue from the chronic toxicity testing of Triphala in male rats. All histological sections of the brain, lungs (hematoxylin and eosin staining, 10×), heart, liver, kidneys, and pancreas (hematoxylin and eosin staining, 40×) of rats.

Interestingly, the tissue sections of the brain, lung, heart, liver, kidney, and pancreas of female (Figure 2) and male (Figure 3) rats treated with 600, 1200, and 2400 mg/kg of TPL demonstrated normal architecture and insignificant variations in the histological and cellular structures of all organs. When comparing the brains of rats administered TPL to those of rats in the normal control group, no demyelination and spongiosis were observed in the cerebrum and cerebellum. Normal cellular structures were identified in the bronchiole, alveoli, alveolar duct, and blood vessels of the lung. The architecture of cardiac muscle cells and connective tissue was normal in the heart. The cellular structures of hepatocytes, sinusoids, and the central vein in the liver were comparable to those of the control group. For the kidney, epithelial lining glomerular tufts and renal tubules were observed with no abnormalities. Comparatively, no abnormalities of acinar and islet cells were identified in the pancreas of rats following treatment of TPL, as compared to rats in the control group. 

## 4. Discussion

Herbal remedies from traditional medicinal plants are commonly used in the healthcare systems of developing and underdeveloped countries [24,25]. Some people use herbal remedies improperly and carelessly because they believe that all herbal medicines are safe since the remedies are derived from natural sources. Despite the fact that herbal medicines are widely used by healthcare personnel worldwide, there is still a lack of scientific data regarding the toxicities and side effects of certain herbal recipes [26]. According to previous studies, several medicinal plants can lead to a variety of illnesses, organ toxicities, morbidity, and mortality in humans [27]. In order to assure the safety of herbal products for human consumption, toxicity tests in animals and systematic studies are required to review the acute toxicities and define the safe dosage [28]. 

In Ayurveda, a traditional system of medicine, Triphala is a powdered mixture of three fruits, *E. officinalis*, *T. bellirica*, and *T. chebula*, and is used to treat numerous illnesses [3,29]. Each ingredient in Triphala was proven to be safe by acute and/or subacute toxicity testing in animals. For example, the methanolic extract of *E. officinalis* fruit in Swiss albino mice [30] and *T. chebula* hydroalcoholic extracts in BALB/c mice [31]. Furthermore, the aqueous extract from the dried fruits of *T. bellirica* did not cause chronic toxicity in either female or male rats [32]. In this study, we qualify herbs by implementing a control approach established by the Thai Herbal Pharmacopoeia (THP) [33]. Gallic acid and ellagic acid are the most common compounds found in Triphala that are used as standards [34]. However, the active compounds within plants might differ based on a variety of factors, such as the planting location and the cultivar [35]. According to the OECD 425 guideline, the short-term use of TPL is highly recommended because there have never been reports of toxic signs from the short-term use of this recipe [36]. In addition, data suggesting the safety of the long-term use of TPL is insufficient. 

Before the study of the efficacy of herbal products in clinical trials, the testing of acute and chronic toxicities in animals is usually a primary step to determine the appropriate dose and duration of product administration [37]. Considering the dose of Triphala for chronic toxicity tests in rats, the 1200 mg/kg concentration of Triphala was selected as an ideal concentration in this study because it is the dosage at which the anti-inflammatory and anti-nociceptive effects were shown to be effective in a previous study [38]. The doses of 600 mg/kg and 2400 mg/kg were selected as the lowest and highest concentrations to investigate the chronic toxicity of TPL. Based on this research, an acute toxicity test and a 270-day chronic toxicity study were performed on experimental animals to assess the safety profile of an aqueous solution extract of Triphala.

The acute oral toxicity test was used to evaluate the adverse effects following a large dose of drug administration within 24 hours. The use of a single oral administration of TPL at 5000 mg/kg was selected in this study because 2000 mg/kg was preliminarily tested and did not cause any symptoms of toxicity or death. The results demonstrated that a high dose of the aqueous solution of TPL did not result in death or severe organ toxicity. Although a slight difference in body weight between the control and treatment groups was observed, this finding was not statistically significant (Table 1). The internal organs, including the brain, lungs, heart, liver, kidney, spleen, stomach, duodenum, small intestine, and sex organs were weighed, gross, and thoroughly microscopically examined, revealing further that the TPL did not induce tissue damage. Therefore, a single oral dose of TPL was not toxic to rats in terms of body weight or internal organs, with a decrease in sex organs. Testicular weight varies with age in rodents—this is dependent on species, strain, nutrition, and living conditions [39], and the increased testicular weights are related to the growth of testicular cells, with a response in total sperm production rate and seminiferous tube size [40,41]. In the case of chronic toxicity, however, the parameter related to reproductive organs should be carefully evaluated. In addition, further research on the reproductive toxicity of Triphala in rats is considered necessary.

Chronic toxicity tests are carried out for 9 to 12 months and investigate whether repetitive exposure to substances can cause toxic signs that might not exhibit toxicity immediately. According to WHO guidelines, the duration of substance administration in animal testing should be based on the estimated duration of clinical use in humans [37]. Changes in general behaviors and body weight are one of the most important indicators for the onset of substance-induced toxicity. These changes include the signs that animals continue losing their body weight so that they may be unable to survive [32,42,43]. From the results of this study, with the chronic administration of TPL for 270 days, the mean body weights of both male and female rats showed no significant change between the control and TPL-treated groups. It is now confirmed that TPL is safe for long-term use in rats. 

Alterations in internal organ weight have long been recognized as a sensitive marker of drug-induced organ changes. In toxicological experiments, organ weights are compared between the treated and untreated animals [44]. From the results, the assessment of the gross pathology of the TPL-treated groups revealed no significant deformity, and the findings were comparable to those of the control group (Table 3 and Table 4). There were some minor statistically significant changes in the TPL-treated animals, but these values still remained within the normal range [45,46]. In chronic toxicity studies with an increase or decrease in internal organ weight, this may be attributed to the changed body weight of the individual rats, which causes their internal organs to be different from the control [47]. Based on the principles and procedures of OECD Test Guideline 452, we conclude in this study that TPL is non-toxic. However, hematology, blood chemistries, and histopathological examinations on female rats (heart, liver, spleen, adrenal gland, and kidney) and male rats (liver, pancreas, kidney, testis, and epididymis) should be considered intently. 

To determine direct cellular damage to the internal organs, blood chemistry and hematology are applied to look for tissue injury or stress responses. Blood transports numerous nutrients and foreign substances in the body, therefore it is a sensitive target for toxic compounds and an important index of physiological and pathological states [48,49]. As a result, blood components such as red blood cells, white blood cells, platelets, and hemoglobin will be firstly impaired by the toxic substance [50]. According to the hematological and other parameters, regional characteristics and local environmental factors may influence blood profile variations, including temperature [51,52]. In chronic toxicity, hematological parameters varied greatly between extracts, doses, and genders, with the predominant alteration being a decrease in RBC, Hb, and HCT levels, along with a decrease in the MCV index. This clinical condition was observed in males treated with Triphala 2400 mg/kg and satellite groups. However, these alterations lacked a dose-response correlation and may be the consequence of individual variation within the normal range [45,53,54]. Moreover, TPL caused leukopenia, especially in neutrophils. In a previous study, TPL could stimulate neutrophil function without increasing the number of neutrophils in mice [55]. Furthermore, TPL significantly increased in lymphocytes, including cytotoxic T and B lymphocytes [56], which resigned results consistent with this study, but the effect was seen only in male rats.

A clinical blood chemistry analysis was conducted to quantify any toxic effects on pancreatic function (glucose), liver function (AST, ALT, ALP, total protein, albumin, and bilirubin), and renal function (BUN and creatinine). In general, rats with serum glucose exceeding 200 mg/dL are classified as “hyperglycemic” [54,57]. Thus, the significantly higher blood sugar level observed in the TPL-treated male rats is still within normal ranges, suggesting no destruction of pancreatic islets. Additionally, TPL was not directly toxic or the cause of hepatotoxin-induced toxicity in the liver, as shown by the absence of alteration in AST, ALT, and ALP levels [58,59]. Toxic substances can directly access the kidneys because of the high volume of blood that flows through the organ. The renal tubules could become obstructed with a wide variety of toxins. Among the most reliable indications of renal disease are alterations in creatinine and BUN levels [60]. In this study, BUN (in females and males) and creatinine (in females) were significantly changed. However, these values changed marginally and remained within the normal range [45,46,61,62,63]. Therefore, Triphala was not toxic to the kidneys. In addition, total protein levels in the blood are a preliminary indicator of normal protein status in the normally functioning kidney and liver [64]. Total protein in male rats was slightly decreased but remained within the normal range. The incidence of delayed toxic effects or the possibility of reverse effects was assessed additionally in the satellite group [23,65]. Triphala was given to the rats in the satellite group at a dose of 2400 mg/kg/day for 270 days and the rats were kept alive for 28 more days. Significant increases in glucose and BUN levels were detected, but no hazardous symptoms were found. Interestingly, the elevated level of BUN and creatinine in female rats was within a normal range. Taken together, this recipe is safe for rats according to the chronic toxicity test in satellite rats. These blood chemistry results suggested that the histopathological examination of females (kidney), males (pancreas and kidney), and the male satellite group (pancreas and kidney) should be carefully assessed. From the results of the chronic toxicity tests, including general behaviors, body, internal organ weight, gross pathology, hematology, and blood chemistries, it could be concluded that TPL had no toxic effect in rats. However, the histopathological examination should be further examined to confirm the toxicity of TPL. 

Histopathological examinations of internal organs were conducted on all animals to confirm the safety of TPL. There was no indication of toxicity and no histological abnormalities found in any of the internal organs of the rats that were treated with TPL, especially female rats (heart, liver, spleen, adrenal gland, and kidney), male rats (liver, pancreas, kidney, testis, and epididymis), and the male satellite group (pancreas and kidney). Figure 2 and Figure 3 represent the vital internal organs. From this study, the rats’ dose of Triphala at 2400 mg/kg could be calculated to be a human dose of approximately 23,000 mg/60 kg body weight [66]. Consequently, the chronic toxicity test of TPL was considered safe. 

## 5. Conclusions

In conclusion, a single dose of 5000 mg/kg of oral Triphala extracted in distilled water in a rat model showed no acute toxicity. There were no toxic effects following the chronic oral administration of 600, 1200, and 2400 mg/kg/day of Triphala for 270 days, suggesting the safety of Triphala for long-term use.

## Figures and Tables

**Table 1:** Body weight of rats in acute toxicity test.

Group	Body Weight (g)			Total Weight Gain at Day 14
	Day 0	Day 7	Day 14	
**Female**				
Control	181.00 + 3.78	219.50 + 5.89	239.00 + 4.52	58.00 + 3.00
Triphala 5000 mg/kg	185.00 + 4.01	219.00 + 4.82	245.00 + 3.80	60.00 + 3.87
**Male**				
Control	209.00 + 3.48	274.50 + 3.76	306.50 + 3.42	97.50 + 3.82
Triphala 5000 mg/kg	209.00 + 4.07	272.50 + 3.82	304.00 + 5.57	95.00 + 4.83

Values are expressed as mean + S.E.M, *n* = 10 (female), *n* = 10 (male).

**Table 2:** Organ weight (g) of female and male rats in acute toxicity test.

	Female		Male	
Organs	Control	Triphala (5000 mg/kg)	Control	Triphala (5000 mg/kg)
Brain	1.80 ± 0.05	1.74 ± 0.10	1.79 ± 0.06	1.75 ± 0.05
Lung	1.35 ± 0.13	1.26 ± 0.18	1.63 ± 0.13	1.55 ± 0.16
Heart	0.88 ± 1.37	0.88 ± 0.08	1.08 ± 0.11	1.02 ± 0.07
Liver	6.83 ± 1.37	7.27 ± 1.36	9.47 ± 0.45	9.83 ± 0.71
Spleen	0.77 ± 0.07	0.70 ± 0.15	0.86 ± 0.13	0.88 ± 0.09
Adrenal gland	0.89 ± 0.10	0.84 ± 0.08	1.25 ± 0.08	1.11 ± 0.07
Kidney	0.04 ± 0.01	0.04 ± 0.01	0.03 ± 0.01	0.03 ± 0.01
Ovary/Epididymis	0.07 ± 0.01	0.07 ± 0.01	0.50 ± 0.07	0.47 ± 0.04
Uterus/Testis	0.65 ± 0.13	0.47 ± 0.10 *	1.75 ± 0.04	1.66 ± 0.06 *

Values are expressed as mean + S.E.M, *n* = 10 (female), *n* = 10 (male). * Significant difference from control, *p* < 0.05.

**Table 3:** Body weight of female rats in chronic toxicity test.

Group	Body Weight (g)						Total Weight Gain at Day 270
	Day 1	Day 30	Day 90	Day 180	Day 270	Day 298	
Control	224.00 ± 6.05	287.50 ± 5.34	332.50 ± 6.92	350.50 ± 3.69	331.50 ± 6.15		107.50 ± 8.04
Triphala 600 mg/kg	226.50 ± 6.28	282.50 ± 5.83	326.50 ± 6.06	346.00 ± 7.48	330.00 ± 5.77		103.50 ± 6.24
Triphala 1200 mg/kg	229.00 ± 5.21	292.00 ± 4.23	328.00 ± 6.20	353.00 ± 8.00	336.00 ± 7.02		107.00 ± 7.86
Triphala 2400 mg/kg ^a^	229.50 ± 5.98	281.50 ± 6.10	315.00 ± 6.24	333.50 ± 6.33	325.50 ± 5.24		96.00 ± 5.57
Triphala 2400 mg/kg ^b^	226.50 ± 5.68	270.00 ± 6.32C *	309.50 ± 7.09 *	342.00 ± 4.90	316.50 ± 9.75	323.00 ± 10.22	90.00 ± 8.13

Values are represented as mean + S.E.M, *n* = 10, * Significant difference from control, *p* < 0.05; ^a^: group of rats that received Triphala at 2400 mg/kg/day for 270 days, then were sacrificed. ^b^: group of rats that received Triphala at 2400 mg/kg/day for 270 days, then were observed for 28 days before being sacrificed.

**Table 4:** Body weight of male rats in chronic toxicity test.

Group	Body Weight (g)						Total Weight Gain at Day 270
	Day 1	Day 30	Day 90	Day 180	Day 270	Day 298	
Control	249.50 ± 6.21	408.00 ± 8.37	512.50 ± 9.92	530.50 ± 14.03	496.50 ± 23.54		247.00 ± 22.57
Triphala 600 mg/kg	249.00 ± 3.93	414.50 ± 5.98	509.50 ± 10.34	554.00 ± 7.22	523.50 ± 20.63		274.50 ± 22.61
Triphala 1200 mg/kg	246.00 ± 4.14	418.50 ± 2.11	504.00 ± 5.76	535.50 ± 5.35	562.00 ± 9.89 *		316.00 ± 9.21 *
Triphala 2400 mg/kg ^a^	253.00 ± 7.61	410.50 ± 7.65	484.50 ± 6.43 *	525.00 ± 9.28	523.00 ± 15.26		270.00 ± 13.66
Triphala 2400 mg/kg ^b^	246.00 ± 6.18	409.00 ± 5.81	494.00 ± 11.45	545.50 ± 18.57	537.00 ± 20.93	544.00 ± 27.82	291.00 ± 20.08

Values are represented as mean + S.E.M, *n* = 10, * Significant difference from control, *p* < 0.05; ^a^: group of rats that received Triphala at 2400 mg/kg/day for 270 days, then were sacrificed. ^b^: group of rats that received Triphala at 2400 mg/kg/day for 270 days, then were observed for 28 days before being sacrificed.

**Table 5:** Organ weight of female rats in chronic toxicity test.

Organs	Control	Triphala (mg/kg)			
		600	1200	2400 ^a^	2400 ^b^
Brain	1.96 ± 0.03	1.96 ± 0.03	1.93 ± 0.02	1.90 ± 0.02	1.90 ± 0.02
Lung	2.31 ± 0.27	2.31 ± 0.27	2.29 ± 0.14	2.35 ± 0.26	2.30 ± 0.31
Heart	1.31 ± 0.03	1.31 ± 0.03	1.46 ± 0.05 *	1.37 ± 0.03	1.21 ± 0.04
Liver	10.32 ± 0.31	10.32 ± 0.31	9.77 ± 0.37	9.42 ± 0.29 *	9.38 ± 0.29 *
Pancreas	1.40 ± 0.19	1.40 ± 0.19	1.08 ± 0.09	0.96 ± 0.06	0.92 ± 0.04
Spleen	0.99 ± 0.10	0.73 ± 0.04 *	0.70 ± 50.01 *	0.74 ± 0.04 *	0.72 ± 0.03 *
Adrenal gland	0.04 ± 0.00	0.04 ± 0.00	0.04 ± 0.00	0.04 ± 0.00	0.03 ± 0.00 *
Kidney	1.27 ± 0.02	1.27 ± 0.02	1.29 ± 0.02	1.28 ± 0.02	1.20 ± 0.02 *
Ovary	0.08 ± 0.01	0.06 ± 0.00	0.08 ± 0.02	0.06 ± 0.00	0.05 ± 0.00
Uterus	1.41 ± 0.32	0.94 ± 0.07	1.17 ± 0.13	1.45 ± 0.29	1.33 ± 0.12

Values are represented as mean + S.E.M, *n* = 10, * Significant difference from control, *p* < 0.05; ^a^: group of rats that received Triphala at 2400 mg/kg/day for 270 days, then were sacrificed. ^b^: group of rats that received Triphala at 2400 mg/kg/day for 270 days, then were observed for 28 days before being sacrificed.

**Table 6:** Organ weight of male rats in chronic toxicity test.

Organs	Control	Triphala (mg/kg)			
		600	1200	2400 ^a^	2400 ^b^
Brain	2.05 ± 0.04	1.94 ± 0.06	1.97 ± 0.03	2.01 ± 0.04	1.96 ± 0.04
Lung	3.89 ± 0.30	3.99 ± 0.43	3.22 ± 0.31	2.66 ± 0.22	3.46 ± 0.74
Heart	1.65 ± 0.07	3.17 ± 1.37	1.96 ± 0.05	1.82 ± 0.05	1.89 ± 0.03
Liver	14.75 ± 0.88	14.18 ± 1.48	17.29 ± 0.59	15.46 ± 0.63	18.93 ± 1.19 *
Pancreas	1.29 ± 0.13	1.53 ± 0.13	1.39 ± 0.10 *	1.38 ± 0.15	1.18 ± 0.07
Spleen	0.98 ± 0.05	1.05 ± 0.04	1.26 ± 0.10	0.96 ± 0.03	1.09 ± 0.04
Adrenal gland	0.03 ± 0.00	0.03 ± 0.00	0.03 ± 0.00	0.03 ± 0.00	0.04 ± 0.00
Kidney	1.65 ± 0.03	1.80 ± 0.03 *	1.92 ± 0.03 *	1.68 ± 0.03	2.00 ± 0.05 *
Testis	1.95 ± 0.05	1.95 ± 0.05	2.10 ± 0.04 *	2.13 ± 0.02 *	2.08 ± 0.07
Epididymis	0.80 ± 0.02	0.80 ± 0.03	0.87 ± 0.02	0.92 ± 0.02 *	0.87 ± 0.03

Values are represented as mean + S.E.M, *n* = 10, * Significant difference from control, *p* < 0.05; ^a^: group of rats that received Triphala at 2400 mg/kg/day for 270 days, then were sacrificed. ^b^: group of rats that received Triphala at 2400 mg/kg/day for 270 days, then were observed for 28 days before being sacrificed.

**Table 7:** Hematological parameters of female rats in chronic toxicity test.

Organs	Control	Triphala (mg/kg)			
		600	1200	2400 ^a^	2400 ^b^
RBC (×10^6^/μL)	8.87 ± 0.26	9.03 ± 0.26	8.93 ± 0.16	8.57 ± 0.15	8.76 ± 0.26
HB (g/dL)	15.74 ± 0.42	15.80 ± 0.35	15.90 ± 0.23	15.15 ± 0.21	15.78 ± 0.44
HCT (%)	51.00 ± 1.50	51.90 ± 1.46	51.80 ± 0.87	49.60 ± 0.60	50.10 ± 1.52
MCV (fl)	57.50 ± 0.40	57.30 ± 0.21	57.60 ± 0.31	58.00 ± 0.33	57.20 ± 0.36
MCH (pg)	17.96 ± 0.14	17.51 ± 0.22 *	17.72 ± 0.13	17.75 ± 0.13	18.04 ± 0.15
MCHC (g/dL)	31.25 ± 0.24	30.51 ± 0.35 *	30.73 ± 0.11	30.65 ± 0.17	31.60 ± 0.24
PLT (×10^6^/μL)	0.77 ± 0.04	0.76 ± 0.03	0.81 ± 0.02	0.73 ± 0.02	0.71 ± 0.04
WBC (×10^3^/μL)	3.69 ± 0.29	3.34 ± 0.34	3.73 ± 0.14	3.70 ± 0.19	3.71 ± 0.60
NEU (×10^3^/µL)	0.82 ± 0.10	1.06 ± 0.17	0.79 ± 0.05	0.83 ± 0.18	0.78 ± 0.10
LYMP (×10^3^/µL)	2.80 ± 0.28	2.24 ± 0.25	2.85 ± 0.12	2.79 ± 0.21	2.85 ± 0.54
MONO (×10^3^/µL)	0.08 ± 0.02	0.04 ± 0.01	0.07 ± 0.02	0.06 ± 0.01	0.09 ± 0.02
EO (×10^3^/µL)	0.00 ± 0.00	0.01 ± 0.01	0.01 ± 0.01	0.02 ± 0.01	0.01 ± 0.01
BASO (×10^3^/µL)	0.00 ± 0.00	0.00 ± 0.00	0.00 ± 0.00	0.00 ± 0.00	0.00 ± 0.00

Values are represented as mean + S.E.M, *n* = 10, * Significant difference from control, *p* < 0.05; ^a^: group of rats that received Triphala at 2400 mg/kg/day for 270 days, then were sacrificed. ^b^: group of rats that received Triphala at 2400 mg/kg/day for 270 days, then were observed for 28 days before being sacrificed.

**Table 8:** Hematological parameters of male rats in chronic toxicity test.

Organs	Control	Triphala (mg/kg)			
		600	1200	2400 ^a^	2400 ^b^
RBC (×10^6^/μL)	10.55 ± 0.44	10.10 ± 0.23	9.35 ± 0.28 *	9.67 ± 0.13*	9.86 ± 0.31
HB (g/dL)	17.87 ± 0.62	17.15 ± 0.32	16.18 ± 0.20 *	16.24 ± 0.19 *	16.76 ± 0.53 *
HCT (%)	56.30 ± 1.33	56.30 ± 1.53	51.40 ± 1.50 *	53.00 ± 0.63	55.60 ± 2.02
MCV (fl)	55.90 ± 0.57	55.60 ± 0.40	54.90 ± 0.41	54.70 ± 0.26 *	56.30 ± 0.40 *
MCH (pg)	16.94 ± 0.21	16.97 ± 0.16	17.45 ± 0.57	16.80 ± 0.13	16.97 ± 0.08
MCHC (g/dL)	30.36 ± 0.32	30.49 ± 0.32	31.70 ± 0.98	30.70 ± 0.22	30.20 ± 0.17
PLT (×10^6^/μL)	0.93 ± 0.04	0.92 ± 0.02	0.95 ± 0.06	0.91 ± 0.02	0.89 ± 0.06
WBC (×10^3^/μL)	7.82 ± 0.75	5.67 ± 0.57	6.99 ± 0.33	6.81 ± 0.58	7.65 ± 1.52
NEU (×10^3^/µL)	2.76 ± 0.74	1.34 ± 0.23 *	1.10 ± 0.10 *	1.47 ± 0.44 *	1.25 ± 0.31 *
LYMP (×10^3^/µL)	4.77 ± 0.61	4.18 ± 0.55	5.76 ± 0.30	5.10 ± 0.38	6.15 ± 1.08
MONO (×10^3^/µL)	0.24 ± 0.07	0.14 ± 0.04	0.11 ± 0.02	0.21 ± 0.08	0.20 ± 0.12
EO (×10^3^/µL)	0.02 ± 0.01	0.02 ± 0.01	0.01 ± 0.01	0.02 ± 0.01	0.05 ± 0.04
BASO (×10^3^/µL)	0.00 ± 0.00	0.00 ± 0.00	0.00 ± 0.00	0.00 ± 0.00	0.00 ± 0.00

Values are represented as mean + S.E.M, *n* = 10, * Significant difference from control, *p* < 0.05; ^a^: group of rats that received Triphala at 2400 mg/kg/day for 270 days, then were sacrificed. ^b^: group of rats that received Triphala at 2400 mg/kg/day for 270 days, then were observed for 28 days before being sacrificed.

**Table 9:** Biochemical parameters of female and male rats in the chronic toxicity test.

Organs	Control	Triphala (mg/kg)			
		600	1200	2400 ^a^	2400 ^b^
**Female**					
Glucose (mg/dL)	171.30 ± 13.82	152.60 ± 7.58	145.80 ± 4.14	148.20 ± 7.39	174.20 ± 11.47
BUN (mg/dL)	20.29 ± 0.65	23.29 ± 0.79 *	23.75 ± 1.33 *	24.96 ± 0.84 *	21.07 ± 0.78
Creatinine (mg/dL)	0.66 ± 0.02	0.68 ± 0.01	0.68 ± 0.02	0.72 ± 0.02 *	0.67 ± 0.02
Total protein (g/dL)	6.14 ± 0.13	6.20 ± 0.09	6.09 ± 0.08	6.22 ± 0.11	5.95 ± 0.20
Albumin (g/dL)	3.26 ± 0.05	3.27 ± 0.03	3.23 ± 0.05	3.26 ± 0.05	3.27 ± 0.06
Bilirubin					
Total (mg/dL)	0.15 ± 0.02	0.18 ± 0.03	0.15 ± 0.02	0.12 ± 0.01	0.17 ± 0.03
Direct (mg/dL)	0.10 ± 0.01	0.10 ± 0.01	0.09 ± 0.00	0.08 ± 0.01	0.10 ± 0.00
AST (U/L)	240.80 ± 21.26	228.70 ± 17.38	193.10 ± 23.43	184.80 ± 28.47	324.70 ± 60.11
ALT (U/L)	66.10 ± 7.20	129.10 ± 58.35	61.90 ± 8.26	63.60 ± 10.31	115.70 ± 21.82
ALP (U/L)	36.00 ± 7.23	30.40 ± 0.87	33.10 ± 3.33	31.50 ± 2.64	54.60 ± 18.25
**Male**					
Glucose (mg/dL)	154.40 ± 17.36	171.20 ± 13.68	211.60 ± 9.79 *	162.50 ± 10.26	264.40 ± 23.73 *
BUN (mg/dL)	19.71 ± 1.42	17.97 ± 0.73	18.50 ± 0.49	19.81 ± 1.46	24.77 ± 1.42 *
Creatinine (mg/dL)	0.66 ± 0.02	0.64 ± 0.02	0.63 ± 0.02	0.69 ± 0.02	8.93 ± 8.12
Total protein (g/dL)	6.53 ± 0.27	6.38 ± 0.22	5.91 ± 0.11 *	5.92 ± 0.07 *	5.90 ± 0.18 *
Albumin (g/dL)	2.98 ± 0.06	3.01 ± 0.05	2.95 ± 0.03	3.03 ± 0.03	5.69 ± 2.81
Bilirubin					
Total (mg/dL)	0.15 ± 0.02	0.12 ± 0.02	0.11 ± 0.01	0.10 ± 0.01 *	0.11 ± 0.01
Direct (mg/dL)	0.08 ± 0.01	0.07 ± 0.01	0.08 ± 0.00	0.07 ± 0.01	0.08 ± 0.00
AST (U/L)	229.70 ± 18.06	214.20 ± 28.93	165.92 ± 23.46	168.60 ± 18.23	198.80 ± 23.17
ALT (U/L)	72.30 ± 7.40	96.10 ± 14.72	72.60 ± 9.94	62.20 ± 6.43	106.50 ± 17.64
ALP (U/L)	87.10 ± 14.01	85.20 ± 18.73	59.40 ± 2.41	53.30 ± 2.99	134.10 ± 43.61

Values are represented as mean + S.E.M, *n* = 10, * Significant difference from control, *p* < 0.05. ^a^: group of rats that received Triphala at 2400 mg/kg/day for 270 days, then were sacrificed. ^b^: group of rats that received Triphala at 2400 mg/kg/day for 270 days, then were observed for 28 days before being sacrificed.

## Data Availability

Not applicable.
